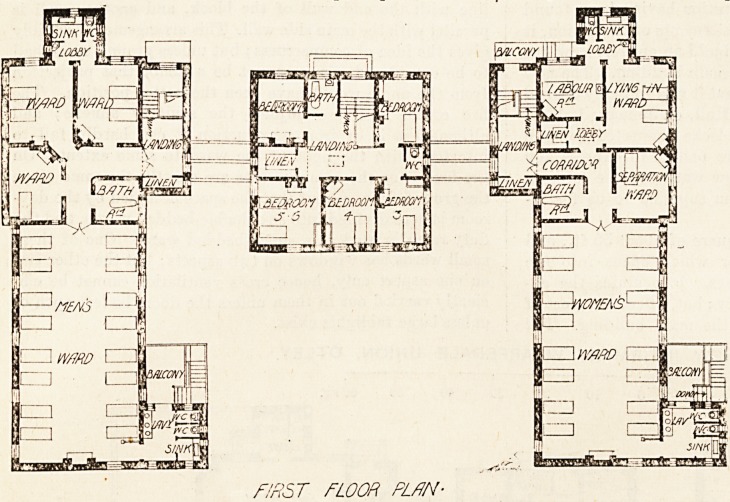# New Infirmary for the Wharfedale Union at Otley, Yorkshire

**Published:** 1907-10-05

**Authors:** 


### NEW INFIRMARY FOR THE WHARFEDALE UNION AT OTLEY, YORKSHIRE.

The old infirmary of this institution having been found to be quite inadequate for the requirements of the Union, it was decided by the Guardians to build an entirely new one in close proximity to the existing main building. The new work was begun in August 1905, and it was formally opened by Mr. F. H. Fawkes, of Fairly Hall, on January 1, 1907. The building consists of three blocks connected to each other by short corridors. We are unable to express any opinion as to the aspect of the new wards, as the cardinal point was not marked on the plan supplied to us for inspection.

The central block is almost a square of about 36 ft., and is divided by the main corridor which opens into the patients' blocks near the staircases. It contains the administrative offices for the infirmary; but, of course, most of the food will be supplied from the main building. The line with the end wall of the block, and another wall is parallel with the main side wall. This arrangement certainly gives the idea of compactness; but unless ground space had to be considered, there cannot be a doubt that projection from the angle would have been the better position. The fire escape staircase adjoins the sanitary annexe; and although it is of iron construction it can hardly fail to interfere with the light of the ward to some extent. On the first floor the arrangements are exactly the same as on the ground floor, except that the space occupied by the day-room is here divided into two double-bedded wards, and the duty-room is used as a single-bedded ward. One of these small wards has windows on two aspects; but the other two on one aspect only, hence cross ventilation cannot be efficiently carried out in them unless the doors be left open or unless large fanlights exist.

NEW INFIRMARY, WHARFEDALE UNION, OTLEY.

GROUND FLOOR PLAN.

ground floor on the right of the hall on entering has the medical officer's room and on the left the head nurse's room. On the other side of the corridor are the scullery, dining-room, and box-room. The first floor contains five bedrooms for the staff, a bath-room, closet, and linen-room.

Passing along the corridor to the men's block, we enter the vestibules, in which are the entrance and staircase on the right, and a little further on is the door of the day-room. This room is 26 ft. long and 16 ft. wide. It is well lighted by a large bay window and two other windows, so that it cannot fail to be a cheerful and pleasant room. Near the centre of the free side of the room is the sanitary block which is provided with a cross-ventilated lobby. Returning to the vestibule, we find the nurses' duty-room and the bath-room. The former is well arranged and well placed, and is supplied with inspection windows from which the large ward can be overlooked; but we do not like the position of the bath-room, as we hold that such adjuncts should never be so much enclosed and should not open from a main thoroughfare corridor. The ward itself is 48 ft. long and 24 ft. wide. It contains 16 beds, arranged in pairs, which is not an uncommon arrangement in workhouse infirmaries, and each pair has a window on each side. The sanitary block projects from the free end of the ward, and one of its walls is in

The first floor of the women's ward is very much like that of the men's ward; but one of the small wards has only one bed, and the space thus set free has been utilised as a much-needed linen-room.

The floors are of fireproof construction, and are then covered with pitch pine blocks one inch and a half in thickness. This makes a good floor, warm, and almost noiseless to walk on, and it will be sanitary also provided the wood does not shrink; but that is just where the difficulty comes in.

The wards have a dado 4 ft. 6 in. high, formed of Parian cement, at the top edge of which and flush with the wall is a string course of glazed bricks; and at the floor level is a cove of glazed brickwork. The bath-rooms are tiled to a height of 6 ft. with white tiles. To prevent the accumulation of dust all the angles throughout the building have been rounded off wherever possible.

The heating is by low pressure steam pipes and radiators; and we are glad to see that open fireplaces also also provided in the wards. Under the main corridor is a subway which contains all the pipes, telephone, and bell wires, etc., so that when repairs are required the flaw can be easily reached and remedied. Tin-lined iron pipes are used for the cold-water service and copper pipes for the hot-water supply. The baths, lavatories, etc., are of enamelled porcelain, and all the fittings would seem to be on the best principle.

FIRST FLOOR PLAN·

The infirmary is built of Guiseley sandstone, and the roofs are covered with green Buttermere slates.

Looking at the infirmary as a whole, it is clearly a well-designed structure for its purpose; and we could wish that the small blots we have noticed did not exist. Furthermore it will be gathered from what we have said that the fitting-up of the infirmary bears no evidence of carelessness or of undue economy, and yet the cost, including furniture, works out at the very modest sum of £108 per bed; and this shows that a good infirmary can even yet be built at a reasonable cost. Of course a General Hospital would cost considerably more, because greater cubic space per bed is required, and many of the adjuncts are much more expensive. The total accommodation of the Otley Infirmary is for 73 patients, and the cost without land was £7,330. The architect was Mr. Herbert Martin, of Bradford; and the principal contractors were Mr. P. Hanan, Mr. Patrick, and Messrs. Suttle and Sons, all of Otley.

## Figures and Tables

**Figure f1:**
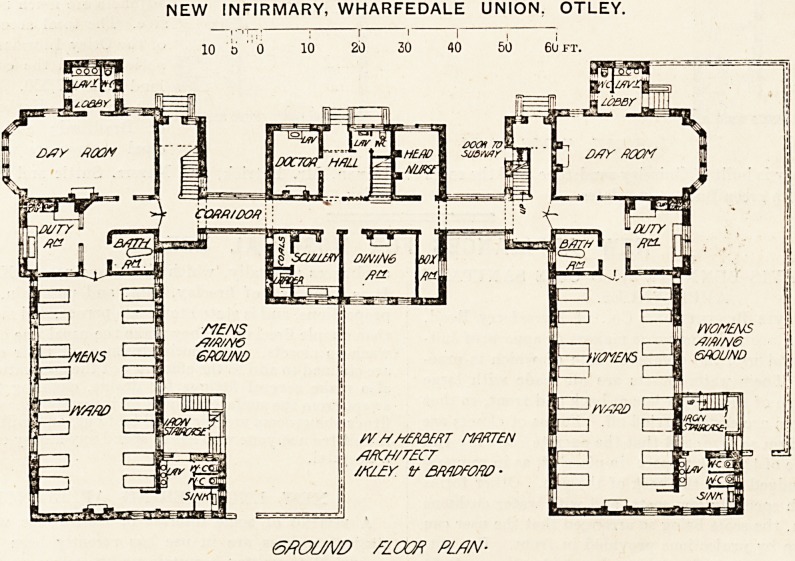


**Figure f2:**